# A Fitness And Lifestyle Intervention Programme (Flip It) - It Is One of Such Interventions Established to Tackle Obesity and Manage Weight Among Inpatients in a Secure Mental Health Service

**DOI:** 10.1192/bjo.2022.391

**Published:** 2022-06-20

**Authors:** David Ho, Raman Deo, Bindu Gurung, Oluwatosin Olabisi, Olufemi Talabi, Maniya Duffy, Kayleigh Reardon

**Affiliations:** 1 Essex Partnership University NHS Foundation Trust, Wickford, United Kingdom; 2 Essex Partnership University NHS Foundation Trust, Basildon, United Kingdom

## Abstract

**Aims:**

A Fitness and Lifestyle Intervention Programme (FLIP IT) is a healthy lifestyle programme, developed for patients identified by their multi-disciplinary team that focusses on helping patients improve their understanding of the benefits of living a healthier life and supporting them to live a healthier life. We evaluate the effectiveness of the FLIP IT programme in tackling obesity and managing the weight of inpatients in a medium secure mental health unit.

**Methods:**

Patients requiring support in managing their physical health from different secure wards were enrolled into the FLIP IT programme following identification by the Multidisciplinary team. Each cycle of the programme was completed over an eleven-week period. Data were collected for a total of seven cohorts of the FLIP IT program. Descriptive analysis was conducted with SPSS. Descriptive statistics, including means, frequencies and proportions were generated. Comparison was done between participants measurements at the start and end of the programme.

**Results:**

A total of 55 participant records from seven cohorts of the FLIP IT program were analysed; 33 (60%) male and 22 (40%) female. Ten participants did not complete the program; discontinuation rate of 18.2%, 7(70%) of which were females and 3(30%) of which were males. There was not much changes in BMI category from start to end (34.10 to 34.14) and in Waist to hip ratio only (0.951 to 0.949) subsequently.

**Conclusion:**

Although, it showed only marginal number of improvement in some categories and no improvement in BMI category, also some patient did withdraw from the project. However, this does not mean that project FLIP IT was not useful at all, as it encouraged some participants to make small everyday changes in secure unit to gain understanding into the importance of their physical health.

